# Autosomal recessive human CTLA-4 deficiency with autoimmune infiltration

**DOI:** 10.70962/jhi.20250269

**Published:** 2026-06-09

**Authors:** Helen C. Su

**Affiliations:** 1 Human Immunological Diseases Section, Laboratory of Clinical Immunology and Microbiology, Division of Intramural Research, National Institute of Allergy and Infectious Diseases, National Institutes of Health, Bethesda, MD, USA

## Abstract

This issue of the *Journal of Human Immunity* features a study (https://doi.org/10.70962/jhi.20250227) reporting the long-awaited first case of autosomal recessive human CTLA-4 deficiency. Here, the features of this patient are compared and contrasted to those in CTLA-4 haploinsufficient patients, patients treated with CTLA-4 inhibitors, and *Ctla4*^*−/−*^ mice.

## Teaser

A report in this issue of *JHI* deepens our understanding of human disease associated with loss of CTLA-4.

In this issue of the *JHI*, Catak and colleagues (1) report a case of a child who had severe immunodysregulation with onset during infancy (Table 1). This patient had chronic diarrhea with poor growth and lymphocytic infiltration in the gastrointestinal tract, which was initially diagnosed as celiac disease, and he developed recurrent immune thrombocytopenic purpura with hepatosplenomegaly. He also had mild recurrent upper respiratory tract infections with hypogammaglobulinemia and poor vaccine titers before immunoglobulin replacement therapy was started. Whole-genome sequencing identified a novel homozygous *CTLA4* hypomorphic missense variant. This substitution of a proline for serine at residue 172 within the conserved transmembrane domain resulted in reduced surface expression and increased lysosomal degradation of CTLA-4 following its internalization from the cell surface. The patient had been previously treated with methylprednisolone and azathioprine, and only later with abatacept was disease adequately controlled. Consistent with the loss of CTLA-4 driving the immunodysregulation in vivo, abatacept normalized the increased activation and proliferation of the patient’s CD4^+^ T cells when they were cocultured in vitro with antigen-presenting cells (APC). Detailed phenotyping before and after abatacept initiation showed normalization of various immune abnormalities, including the increases in circulating activated follicular helper T cells (T_FH_), effector memory and exhausted T cells, IFN-γ and IL-10 expression in T cells, as well as activated and atypical memory B cell subsets. The patient is currently 9 years old and remains clinically well after starting abatacept, with resolution of his hepatosplenomegaly and no recurrence of thrombocytopenia, neutropenia, or diarrhea.

**Table 1.** Comparison of genetic and/or pharmacologic CTLA-4 loss in humans and mice

Disease feature	Human			Mouse	
	*CTLA4* ^*−/−*^	*CTLA4* ^*+/−*^	Ipilimumab-treated	*Ctla4* ^*−/−*^	*Ctla4* ^*+/−*^
Genetic variant	Homozygous Ser172Pro (hypomorphic, *n* = 1)	Heterozygous loss-of-function	Not applicable	Homozygous loss-of-function	Heterozygous loss-of-function
Outcome	Alive at 9 years old on abatacept, immunoglobulin replacement, and prophylactic antibiotics	Variable disease penetrance (∼70% affected). Most respond to sirolimus or abatacept and replacement immunoglobulin if needed. Hematopoietic stem cell transplantation is an option for severe cases	Immune-related adverse effects appear within a few weeks to months after treatment. Most resolve upon delaying dose or with immunosuppressants	Death by ∼3–7 wk of age	Not reported (healthy)
Lymphoproliferation	Hepatosplenomegaly	Lymphadenopathy, hepatosplenomegaly	None	Hepatosplenomegaly	None (healthy)
Lymphocytic infiltration	Enteropathy (celiac disease)	Enteropathy, inflammatory bowel disease, granulomatous-lymphocytic interstitial lung disease, lymphocytic infiltrations in the brain, bone marrow, kidney, atopic dermatitis, and arthritis > pancreas	Dermatitis, enterocolitis, hepatitis, and pneumonitis	Multiorgan mononuclear infiltrates in heart (with cardiomegaly), pancreas, lungs, salivary glands, liver, bone marrow > synovium, and blood vessels	None (healthy)
	​	​	​	Increased activated T cells in spleen, lymph nodes, thymus, and increased activated B cells in spleen and lymph nodes	​
Autoimmunity	Immune thrombocytopenic purpura	Immune thrombocytopenic purpura, autoimmune hemolytic anemia, autoimmune neutropenia, thyroiditis/hypothyroidism, type 1 diabetes mellitus, psoriasis, vitiligo, and alopecia	Hypophysitis with pituitary autoantibodies, thyroiditis/hypothyroidism, and rarely autoimmune cytopenias	Anti-insulin, gastric parietal, Ro52, and dsDNA autoantibodies (when Ctla4 is conditionally deleted in Treg)	None (healthy)
	​	​	​	Worsened collagen-induced arthritis disease, but delayed or ameliorated experimental allergic encephalitis (when Ctla4 is conditionally deleted in Treg)	​
Infection susceptibility	Mild recurrent upper respiratory tract infections	Recurrent sinopulmonary (±bronchiectasis) and otitis media infections	Infrequent	None reported	None (healthy)
​	Elevated EBV DNA copy number (while on methylprednisolone, azathioprine)	​	​	​	​
Other	​	Lymphoma, gastric cancer	​	​	​
Laboratory abnormalities	Increased circulating Tfh, effector memory and exhausted T cells	Normal or low numbers of total lymphocytes, T cells, CD4^+^ T cells, CD8^+^ T cells, T_reg_, B cells, and NK cells	Not investigated/reported	Spontaneous and TCR-stimulated proliferation and cytokine production	Increased germinal centers after immunization
	Increased IFN-γ and IL-10 expression in T cells	Decreased numbers of T_reg_ having impaired suppressor function, naïve T cells, and memory B cells	​	Increased IFN-γ, IL-4, and GM-CSF upon TCR stimulation	​
	Increased activated and atypical memory B cells	Increased numbers of activated memory T cells and circulating T_FH_ cells		Increased Treg, Tfh, and Tfr (but Tfr are functionally impaired)	
	Hypogammaglobulinemia, poor vaccine titers	Hypogammaglobulinemia, poor vaccine titers		Hypergammaglobulinemia	
References	This article in *JHI *(1)	(2, 3, 4, 5)	(2)	(6, 7, 8)	(7, 9)

Cytotoxic T lymphocyte-associated protein 4 (CTLA-4, also known as CD152) plays a crucial role in dampening T cell functions to maintain lymphocyte homeostasis (reviewed in 2, 10). When T cells become activated, CTLA-4 expression translocates to the cell surface, where it can bind CD80/CD86 on APC. Binding of CTLA-4 with CD80/CD86 leads to the latter’s internalization by trans-endocytosis. The resulting depletion of CD80/CD86 molecules on the APC impairs the ability of the APC to deliver costimulatory signals via CD80/CD86 binding of CD28 on the T cells. Because CTLA-4 has a higher affinity than CD28 for CD80/CD86, it outcompetes CD28, thereby inhibiting T cell activation signals to limit T cell proliferation and T cell IL-2 production.

Much of what we know about how CTLA-4 functions came initially from studies of *Ctla4*-deficient (*Ctla4*^*−/−*^) mice, which, similarly to the abovementioned CTLA-4-deficient patient, develop an early-onset multiorgan lymphocytic infiltration (Table 1) (6, 7). Of note, mice rendered genetically deficient for Ctla-4, specifically within regulatory T cells (T_reg_), develop a less severe form of disease, suggesting that loss of constitutive expression of CTLA-4 on T_reg_ cells, along with loss of inducible CTLA-4 expression on activated conventional T cells, both contribute to pathogenesis (8). Overall, mechanistic studies in mice indicate that CTLA-4 functions dually in T_reg_ and conventional effector T cells, which cooperate to maintain immune homeostasis in vivo.

Understanding how CTLA-4 functions in mice provided a rationale for the development of blocking antibodies against CTLA-4, such as ipilimumab, to enhance T cell activity against cancers. In a pivotal trial, ipilimumab improved overall survival in patients with advanced melanoma, which led to the development of other forms of immune checkpoint inhibitors. However, treatment with CTLA-4 inhibitors is associated with severe dose-dependent inflammation, often involving the gastrointestinal tract, liver, skin, or endocrine organs, and autoimmunity when present can be life-threatening (Table 1) (2). These presentations are reminiscent of the *Ctla4*^*−/−*^ mice, although the prevalence, organs predominantly affected, and associated autoimmunity differ somewhat, which might reflect the doses of CTLA-4 inhibitors used and their administration in adulthood as opposed to complete gene deficiency since birth (Table 1).

Interestingly, mice heterozygous for *Ctla4* null alleles do not develop disease (7). Thus, the discovery of disease in humans haploinsufficient for *CTLA4* was surprising (Table 1) (11, 12), although prior studies had shown associations of increased risk for various autoimmune diseases with certain polymorphisms in *CTLA4* or, alternatively, decreased expression of CTLA-4 on T cells, including T_reg_ cells (10). Disease in CTLA-4 haploinsufficient humans displays variable penetrance, with ∼60% of carriers affected and ∼40% of carriers remaining healthy (3). Several hundred affected patients have been reported in the literature, with lymphoproliferation, autoimmunity, and recurrent infections predominating (2, 3, 4, 5). Typical features are lymphocytic infiltrations in the gastrointestinal tract and brain, granulomatous-lymphocytic interstitial lung disease, lymphadenopathy, and splenomegaly. Some patients develop lymphomas or gastric cancers (4). Disease also features autoimmunity, typically manifesting as cytopenias, enteropathy, endocrinopathies such as type I diabetes mellitus and thyroiditis, and skin disorders such as psoriasis and vitiligo. A central role of CTLA-4 in disease pathogenesis is supported by the use of engineered fusion proteins (CTLA4-Ig) such as abatacept that treat disease not only in the CTLA-4 haploinsufficient patients but also in other patients with autoimmune diseases; however, more definitive results establishing the efficacy of such targeted therapy for CTLA-4-haploinsufficient patients await the results of the ongoing phase 2 ABACHAI clinical trial (10). By binding to CD80/CD86, CTLA4-Ig blocks CD80/CD86 engagement with CD28 to thereby interrupt delivery of costimulatory signals to the responding T cell. Finally, CTLA-4 haploinsufficiency in humans also features hypogammaglobulinemia with poor vaccine titers and recurrent infections as a part of their complex immunodysregulatory disorder (3).

Consistent with their clinical findings, Ctla-4-deficient mice exhibit increased numbers of activated T and B cells in spleen and lymph nodes as well as hypergammaglobulinemia (6, 7, 8). The increased B cell responses result from increases in T_reg_, T_fh_ that promote B cell responses, and T follicular T_reg_ that are compromised in their ability to suppress B cell responses (8, 13, 14, 15). By contrast, most CTLA-4 haploinsufficient patients show normal immunological parameters in the peripheral blood, although some patients have decreased numbers of total lymphocytes, T cells, CD4^+^ T cells, CD8^+^ T cells, T_reg_, B cells, and NK cells as well as hypogammaglobulinemia (3). The reason for the hypogammaglobulinemia in CTLA-4 haploinsufficient humans but not in mice is unclear but has been proposed to reflect poor survival of plasma cells due to activated T cell infiltration into the bone marrow (12). More extensive immunological analyses in the peripheral blood of CTLA-4 haploinsufficient patients also show trends of decreased numbers of T_reg_ having impaired suppressor function, naïve T cells, and memory B cells but increased numbers of memory T cells and circulating T_FH_ (5, 16). Of note, treatment with abatacept normalized T_FH_ numbers correlating with improved disease control, although effects on other cell subsets have not been as systematically investigated (5, 16).

Why mice heterozygous for *Ctla4* null alleles remain healthy while heterozygous CTLA-4 haploinsufficient humans do not is unclear, but this disparity could reflect other factors such as HLA/genetic background and/or environment such as microbiome/microbial exposure. Because of their apparent health, mice heterozygous for *Ctla4* null alleles have not been studied in-depth, although a close examination has revealed an increased germinal center area after primary immunization with superantigen Staphylococcal enterotoxin B, suggesting the possibility of other mild cellular abnormalities (9). Regardless, results from mice suggest that humans with monogenic biallelic CTLA-4 deficiency would also develop a similar severe immunodysregulatory disease. This indeed appears to be supported by Catak and colleagues’ first reported case of autosomal recessive CTLA-4 deficiency.

The identification of this case helps us better understand the overall role of CTLA-4 in humans, as well as related disorders such as LRBA or DEF6 deficiencies, which decrease CTLA-4 cell surface expression by impairing its intracellular trafficking. It will be important to identify additional patients to determine whether findings for this index patient can be generalized, including whether abatacept treatment is sufficient to avoid hematopoietic stem cell transplantation. Furthermore, future studies in either CTLA-4 haploinsufficient or additionally CTLA-4 deficient humans should help us better understand the variable disease penetrance and impaired B cell responses that are features of the humans but not their corresponding mutant mouse models.
